# Prognostic utility of the CALLY index in metastatic melanoma: building a nomogram for Patients on Anti-PD-1 therapy

**DOI:** 10.1007/s12094-025-03888-z

**Published:** 2025-03-16

**Authors:** Caner Acar, Haydar Çağatay Yüksel, Gökhan Şahin, Fatma Pinar Açar, Damla Gunenc, Burçak Karaca

**Affiliations:** 1 Division of Medical Oncology, Department of Internal Medicine, Ege University Medical Faculty, 35100 Izmir, Turkey; 2 Division of Medical Oncology, Hatay Training and Research Hospital, 3100 Hatay, Turkey

**Keywords:** CALLY index, Metastatic melanoma, Immune checkpoint inhibitors, Anti-PD-1 therapy, Prognostic biomarker, Survival analysis, Nomogram, Immunonutritional marker

## Abstract

**Background:**

Despite the success of immune checkpoint inhibitors (ICIs) in metastatic melanoma, many patients fail to derive meaningful benefit, underscoring the urgent need for accessible prognostic biomarkers. The C-reactive protein (CRP)–albumin–lymphocyte (CALLY) index, an immunonutritional index, has shown prognostic value in various cancers. Previous studies indicate that systemic inflammation and nutritional status influence ICI efficacy, suggesting the potential relevance of the CALLY index in metastatic melanoma. This study evaluates the CALLY index’s role in metastatic melanoma patients treated with anti-PD-1 therapy.

**Methods:**

This retrospective study analysed 92 patients with metastatic melanoma who were treated with anti-PD-1 monotherapy at Ege University’s Faculty of Medicine between 2015 and 2023. The CALLY index was calculated using the pre-treatment CRP, albumin and lymphocyte levels. Kaplan–Meier analysis was used to estimate survival outcomes, and univariate and multivariate Cox regression models were employed to identify independent prognostic factors. A predictive nomogram incorporating the CALLY index and other significant variables was then developed.

**Results:**

The optimal CALLY index cutoff was determined to be 2. Patients with a low CALLY index (≤ 2) had worse median overall survival (OS) and progression-free survival (PFS) when compared with those who had a high CALLY index (> 2) (median OS: 9.6 vs 31.3 months, p < 0.001; median PFS: 3.8 vs 10.6 months, p = 0.001). Multivariate analysis identified the CALLY index, lactate dehydrogenase above the upper limit of normal, Eastern Cooperative Oncology Group score ≥ 2, M1c/M1d staging and acral/mucosal melanoma subtypes to be independent predictors of OS. A nomogram was then constructed based on these factors, yielding a concordance index of 0.705 (95% confidence interval: 0.634–0.776). This model stratified patients into low-, intermediate- and high-risk groups, with the high-risk group showing significantly worse OS than the intermediate- and the low-risk groups (p < 0.001).

**Conclusion:**

The CALLY index is a cost-effective and independent prognostic biomarker that can aid in risk stratification and guide treatment decisions in patients with metastatic melanoma receiving anti-PD-1 therapy.

**Supplementary Information:**

The online version contains supplementary material available at 10.1007/s12094-025-03888-z.

## Introduction

Immune checkpoint inhibitors (ICIs) are a cornerstone of malignant melanoma treatment. The combination of anti-PD-1 and anti-CTLA-4 inhibitors offers high efficacy, albeit in association with significant toxicity [1]. Despite its relatively low efficacy, anti-PD-1 monotherapy is considered a viable option in patients with good prognostic profiles due to its lower toxicity. Patients with BRAF wild-type tumours, absence of brain and/or liver metastases, and a low tumour burden are particularly suitable candidates for anti-PD-1 monotherapy [2–4] In BRAF-mutant patients, the combination of BRAF and MEK inhibitors, following the findings of DREAMseq trial, has become a less prioritised treatment option. While BRAF/MEK inhibitors achieve high objective response rates, the long-term overall survival tends to favour patients treated with ICIs [5].

While anti-PD-1 and anti-CTLA-4 therapies, either alone or in combination, achieve high objective response rates and good response durations, many patients fail to benefit from ICI treatment. Hence, identifying prognostic biomarkers is essential for optimising treatment outcomes in patients with melanoma who receive ICIs [6]. Additionally, baseline biomarkers capable of predicting high survival rates with anti-PD-1 monotherapy are needed to guide the choice between monotherapy and combination ICI treatment. To date, no validated biomarker has been identified to help clinicians determine which patients with advanced melanoma are more likely to benefit from ICI therapy, although emerging tools, such as the interferon gamma (IFN-γ) signature and circulating tumour DNA (ct DNA), hold promise for improving the response prediction and patient stratification. However, the high cost, limited availability and lack of standardisation of such tools emphasise the need for more accessible and practical markers for use in clinical settings [7].

Both cancer-related inflammation and poor nutritional status promote tumourigenesis and play an important role in ICI resistance. Inflammation promotes an immunosuppressive tumour microenvironment marked by the presence of regulatory T-cells, macrophage polarisation and elevated proinflammatory cytokines, all of which impair T-cell activity and play a role in ICI resistance [8]. Moreover, while the impact of poor nutritional status on the efficacy of ICIs is not as well established as its effect on cancer prognosis, it may influence ICI efficacy through mechanisms such as the increased clearance of monoclonal antibodies and the suppression of the adaptive immune system via enhanced endogenous steroid synthesis, thereby fostering ICI resistance [9]. Consequently, biomarkers reflecting the immunonutritional status may serve as predictive indicators of the treatment responses and survival outcomes in patients with melanoma who are undergoing ICI therapy [10].

Various immunonutritional indices, such as the prognostic nutritional index (PNI) and the modified Glasgow prognostic score (mGPS), have been explored as potential prognostic biomarkers across different malignancies. However, these indices often focus on a narrower subset of parameters—such as serum albumin and total lymphocyte count (PNI) or C-reactive protein and hypoalbuminaemia (mGPS)—and may not fully capture the complexities of the tumour–immune interplay [11]. As a result, there is growing interest in more integrative markers that reflect both systemic inflammation and nutritional status.

The C-reactive protein (CRP)–albumin–lymphocyte (CALLY) index is a novel immunonutritional index that combines the CRP, albumin and lymphocyte counts. By integrating these parameters, the CALLY index reflects a patient’s nutritional, inflammatory and immune status. It is a low-cost and readily accessible tool, which was first shown to have prognostic value in patients with hepatocellular carcinoma [12]. However, to date, no studies have assessed the prognostic role of the CALLY index in patients with melanoma treated with ICIs. Hence, this study aims to evaluate the relationship between the CALLY index scores, treatment responses and survival outcomes in patients with advanced melanoma who are receiving anti-PD-1 therapy.

## Methodology

This retrospective single-centre study included patients who were treated at Ege University’s Faculty of Medicine between March 2015 and November 2023. Data were obtained from the patients’ electronic medical records. A flowchart illustrating the inclusion of patients in the study is provided in Figure S1. The inclusion criteria for the study were as follows: patients diagnosed with metastatic melanoma, treated with anti-PD-1 monotherapy, had pre-treatment CRP, albumin and lymphocyte levels available for calculating the CALLY index and had sufficient follow-up data for outcome assessment. The CALLY index was determined using the following formula: CALLY index = albumin level (g/dL) × lymphocyte count (/μL) / CRP level (mg/dL) × 10.^4^

Anti-PD-1 monotherapy consisted of either nivolumab (n = 87) at 3 mg/kg every 2 weeks or pembrolizumab (*n* = 5) at 200 mg intravenously every 3 weeks. The objective response rate (ORR) was defined as the percentage of patients who achieved either complete or partial remission. Progression-free survival (PFS) was measured from the initiation of ICI therapy to the date of documented disease progression, as assessed using the response evaluation criteria in solid tumours (RECIST) guidelines (version 1.1), or death from any cause. Overall survival (OS) was defined as the time from the initiation of ICI therapy to death from any cause. Moreover, patients who were alive at the end of the study period were censored at the date of their last known contact.

The categorical variables were reported as frequencies and percentages (n, %), while the continuous variables were summarised as minimum, maximum and median values. Comparisons between the low and high CALLY index groups were conducted using appropriate statistical tests—namely, the Chi-square or Fisher’s exact test for the categorical variables and Mann–Whitney U test for the continuous variables.

Both PFS and OS were estimated using the Kaplan–Meier method, with the differences between groups being assessed using the log-rank test. The predictive factors for PFS and OS were evaluated using the Cox proportional hazards regression analysis. Logistic regression analysis was used to identify factors associated with the ORR. The optimal cutoff values for the CALLY index and other variables were determined using the survminer package in R (version 4.4.2; accessed on 5 November 2024), which applies an outcome-oriented approach (the maximally selected rank statistic) to identify the cutoff that best discriminates survival outcomes between groups. The nomogram construction was performed using the rms package in R. All of the other statistical analyses were performed using jamovi (version 2.3.28, the jamovi project, 2023, https://www.jamovi.org). A p-value of < 0.05 was considered to be statistically significant.

## Results

### Baseline characteristics

Of the 128 patients with metastatic melanoma who were treated with anti-PD-1 therapy at Ege University’s Faculty of Medicine between March 2015 and November 2023, and who had sufficient data to calculate the CALLY index and necessary follow-up information, 92 patients were included in this study. The median OS was 16.9 months (95% confidence interval [CI], 11.8–25.1), while the median PFS was 5.4 months (95% CI, 4–9.6). As of the cutoff date for the analysis (7 October 2024), 71 patients (77.2%) had died. The ORR was 30.2%.

### Comparison of baseline characteristics between the high and low CALLY index groups

Using the survminer package in R (version 4.4.2; accessed on 5 November 2024), the cutoff value for the CALLY index was determined to be 2, as based on the median OS. To assess the robustness of the CALLY index cutoff of 2, we performed an internal validation using bootstrap resampling (1000 iterations). The log-rank p-value for OS remained significant (*p* = 0.005), indicating a stable cutoff within our cohort. On that basis, the patients were classified into two groups: low CALLY (≤ 2) and high CALLY (> 2). Of the 92 participating patients, 50 (54.4%) were in the low CALLY group, whereas 42 (45.6%) were in the high CALLY group. The baseline characteristics in the two groups are summarised in Table 1. First-line anti-PD-1 therapy was more frequent in the high CALLY group (71.4% vs 50%, *p* = 0.037), while the median lactate dehydrogenase (LDH) level in that group was significantly lower (191 U/L vs 275 U/L, *p* < 0.001). No statistically significant differences were found in terms of the other baseline characteristics.

**Table 1:** Comparison of baseline characteristics between CALLY Low and high groups

	Total (*n* = 92)	CALLY $\le 2$ (*n* = 50)	CALLY > 2 (*n* = 42)	*p*
Age, years (median)	62 (30–80)	61.5 (32–80)	62 (30–80)	0.441
Gender				
Female	39 (42.4%)	22 (44%)	17 (40.5%)	0.733
Male	53 (57.6%)	28 (56%)	25 (59.5%)	
Histological subtype				
Nodular	15 (16.3%)	6 (12%)	9 (21.4%)	0.335
Superficial Spreading	18 (19.6)	10 (20%)	8 (19%)	
Acral Lentiginous	14 (15.2)	11 (22%)	3 (7.1%)	
Mucosal	4 (4.3%)	2 (4%)	2 (4.8%)	
Uveal	6 (6.5%)	2 (4%)	4 (9.5%)	
Others	35 (38%)	19 (38%)	16 (38.1%)	
BRAF V600 status				
Wild	68 (73.9%)	35 (70%)	33 (78.6%)	0.351
Mutant	24 (26.1%)	15 (30%)	9 (21.4%)	
Stage				
M1a	26 (28.3%)	10 (20%)	16 (38.1%)	0.091
M1b	18 (19.6%)	8 (16%)	10 (23.8%)	
M1c	34 (37%)	22 (44%)	12 (28.6%)	
M1d	14 (15.2%)	10 (20%)	4 (9.5%)	
ECOG status				
0–1	75 (81.5%)	39 (78%)	36 (85.7%)	0.342^+^
≥ 2	17 (18.5%)	11 (22%)	6 (14.3%)	
ICI treatment line				
1	55 (59.8%)	25 (50%)	30 (71.4%)	**0.037**
≥ 2	37 (40.2%)	25 (50%)	12 (28.6%)	
Previous BRAF + MEK inhb				
No	74 (80.4%)	37 (74%)	37 (88.1%)	0.090
Yes	18 (19.6%)	13 (26%)	5 (11.9%)	
Previous Chemotherapy				
No	75 (81.5%)	38 (76%)	37 (88.1%)	0.137
Yes	17 (18.5%)	12 (24%)	5 (11.9%)	
LDH, U/L	218 (113–3656)	275 (120–3656)	191 (113–528)	** < 0.001**

Data are presented as median (min–max) for continuous variables and as n (%) for categorical variables

*ECOG* Eastern cooperative oncology group, *ICI* Immune checkpoint inhibitor, *LDH* Lactate dehydrogenase

### Survival analysis

**Fig. 1:**
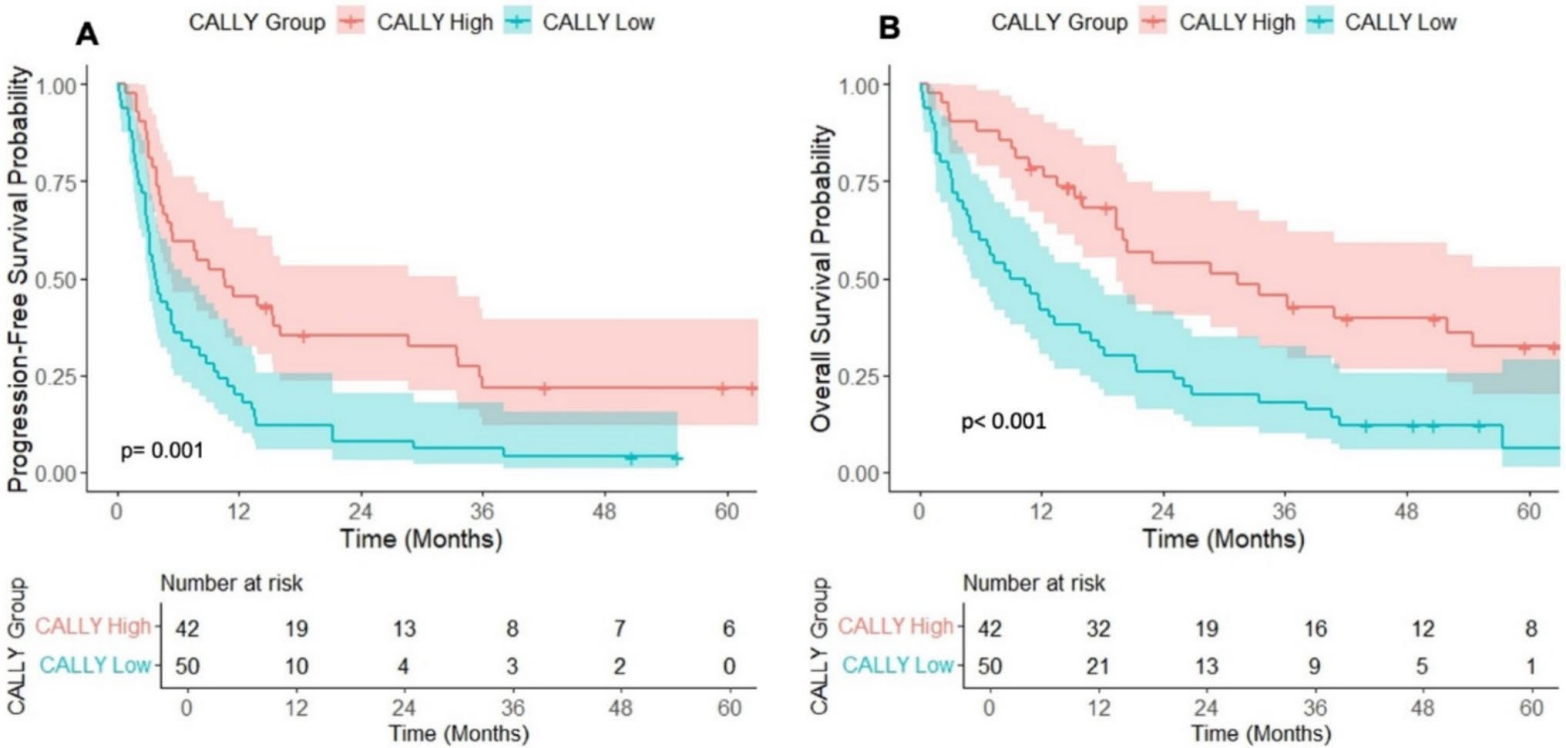
Kaplan–Meier Curves with Log-Rank Test Results for CALLY High and Low Groups: (A) Progression-Free Survival, (B) Overall Survival

The Kaplan–Meier and log-rank tests were used to assess the PFS and OS in the low and high CALLY groups. The median PFS was 3.8 months (95% CI, 3.1–7.3) in the low CALLY group and 10.6 months (95% CI, 5.3–33.5) in the high CALLY group (Fig. 1A). This difference was statistically significant (log-rank, p = 0.001). Similarly, the median OS was 9.6 months (95% CI, 5.1–17.7) in the low CALLY group and 31.3 months (95% CI, 19.4–NA) in the high CALLY group (Fig. 1B), with a statistically significant difference again being observed (log-rank, p < 0.001).

### Prognostic factors for objective response, PFS and OS

The association between the patients’ baseline characteristics, the CALLY index and the objective response is summarised in Table 2. Univariate logistic regression analysis identified the disease stage, LDH above the upper limit of normal (ULN) and the CALLY index as significant predictors. Multivariate analysis revealed the M1c stage to be an independent predictive factor in relation to the objective response (*p* = 0.046), while a low CALLY index was a borderline significant predictive factor (*p* = 0.050).

**Table 2:** Univariate and Multivariate Analyses of Variables Associated with Objective Response

	Univariate OR	95% CI	P	Multivariate OR	95% CI	*P*
Age, years (continuous)	1.000	0.972–1.04	0.847			
Gender						
Male						
Female	0.980	0.423–2.27	0.963			
BRAF V600 status						
Mutant						
Wild	0.980	0.381–2.52	0.967			
Histological subtype						
Superficial Spreading						
Nodular	0.533	0.133–2.14	0.375			
Acral Lentiginous	0.320	0.191–1.89	0.133			
Mucosal	0.267	0.023–3.08	0.290			
Uveal	0.400	0.058–2.77	0.353			
Others	0.600	0.191–1.89	0.382			
Stage						
M1a						
M1b	0.733	0.219–.2.45	0.615	0.515	0.135–1.957	0.330
M1c	0.306	0.104–0.89	0.030	0.309	0.097–0.981	0.046
M1d	0.293	0.072–1.18	0.085	0.336	0.075–1.489	0.151
ECOG status						
0–1						
≥ 2	0.733	0.245–2.19	0.578			
Previous BRAF + MEK inhb						
No						
Yes	1.172	0.415–3.32	0.763			
Previous Chemotherapy						
No						
Yes	0.371	0.111–1.24	0.118			
ICI treatment line						
1						
≥ 2	0.650	0.275–1.53	0.325			
LDH level						
Normal						
Elevated	0.412	0.174–0.980	0.045	0.547	0.204–1.466	0.230
CALLY index						
High						
Low	0.292	0.122–0.695	0.005	0.392	0.153–1.001	0.050

Logistic regression analysis was used to identify variables associated with objective response. Significant variables in univariate analysis (p < 0.1) were included in the multivariate analysis. Results are presented as odds ratios (OR) with 95% confidence intervals (CI). *ECOG* Eastern cooperative oncology group, *LDH* Lactate dehydrogenase, *OR* Odds ratio, *CI* Confidence interval

**Fig. 2:**
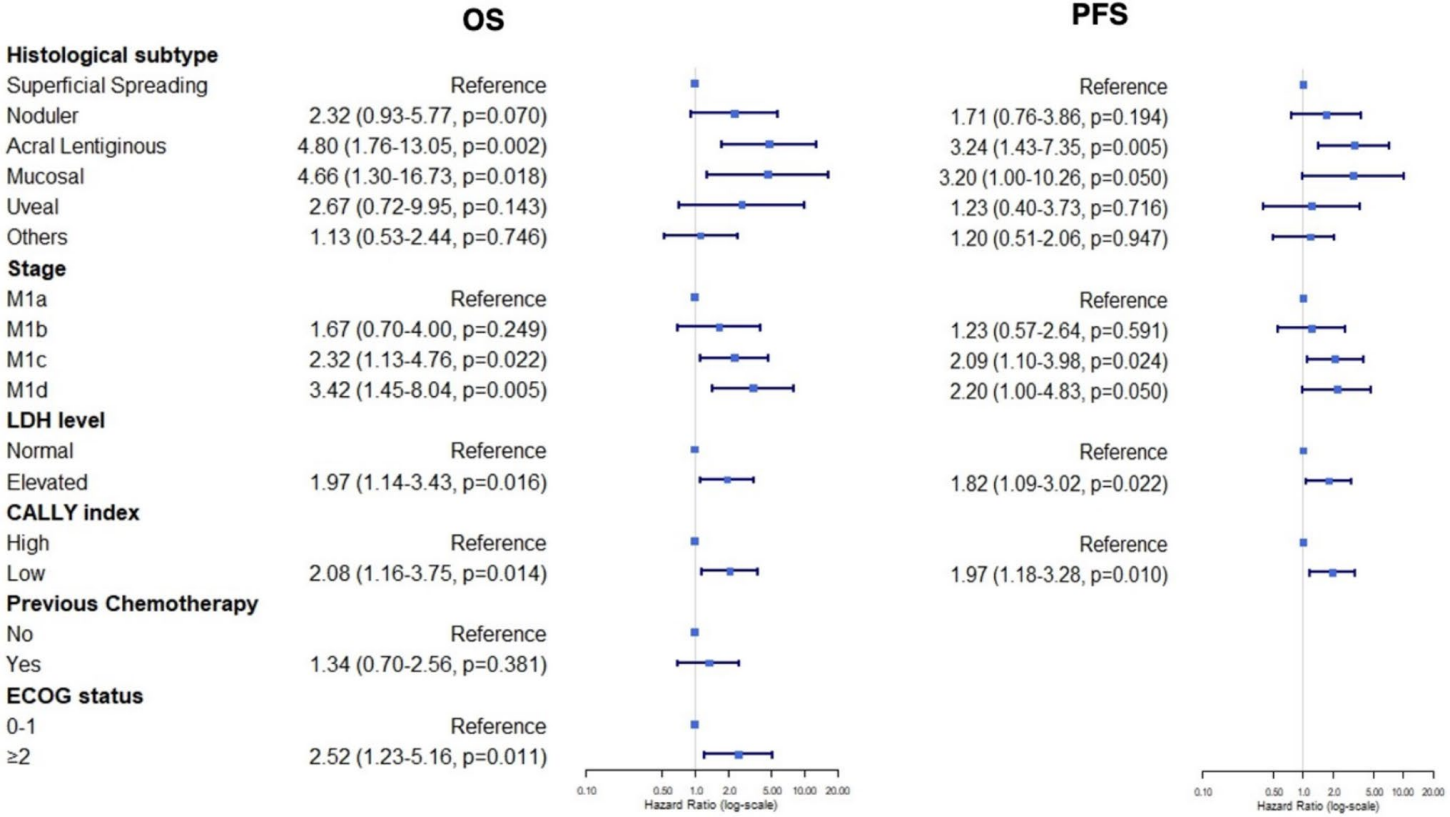
Multivariate analysis of variables for OS and PFS

The results of the univariate and multivariate Cox regression analyses evaluating the predictors of PFS and OS are presented in Tables 3 and Fig. 2. Variables with a *p* < 0.1 in the univariate analysis were included in the multivariate models. Based on the multivariate analysis results, in terms of the OS, the acral lentiginous (*p* = 0.002) and mucosal histological subtypes (*p* = 0.018), M1c (*p* = 0.022) and M1d stages (*p* = 0.005), an Eastern Cooperative Oncology Group (ECOG) score ≥ 2 (*p* = 0.011), LDH > ULN (*p* = 0.016) and a low CALLY index score (*p* = 0.014) were identified as independent risk factors. With regard to the PFS, the acral lentiginous subtype (*p* = 0.005), M1c stage (*p* = 0.024), LDH > ULN (*p* = 0.022) and a low CALLY index score (*p* = 0.010) were significant independent predictors. Although line of therapy did not meet the *p* < 0.1 threshold for inclusion in our final multivariate model, we tested an alternative model that included line of therapy, given that first-line anti-PD-1 treatment was more frequent in the high CALLY group. Including line of therapy did not alter the prognostic significance of the CALLY index, indicating that differences in treatment lines did not substantially bias our findings.

**Table 3:** Univariate analysis of variables associated with PFS and OS

	OS			PFS		
	HR	95% CI	*P*	HR	95% CI	*p*
Age (continuous), years	1.01	0.99–1.03	0.252	1.00	0.99–1.02	0.845
Gender						
Male						
Female	1.16	0.73–1.86	0.524	1.13	0.73–1.75	0.594
BRAF V600 status						
Mutant				1.20	0.73–1.98	0.470
Wild	1.02	0.40–2.62	0.967			
Histological subtype						
Superficial Spreading						
Nodular	1.48	0.65–3.37	0.348	1.51	0.70–3.28	0.295
Acral Lentiginous	2.72	1.22–6.08	0.014	2.75	1.27–5.94	0.010
Mucosal	1.99	0.64–6.24	0.236	1.85	0.60–5.69	0.285
Uveal	1.53	0.49–4.79	0.465	1.31	0.46–3.70	0.611
Others	1.24	0.63–2.46	0.532	1.37	0.71–2.62	0.347
Stage						
M1a						
M1b	1.33	0.63–2.79	0.454	1.05	0.53–2.07	0.888
M1c	1.78	0.98–3.25	0.058	1.84	1.05–3.22	0.035
M1d	2.60	1.23–5.50	0.012	1.70	0.83–3.47	0.144
ECOG status						
0–1						
≥ 2	1.80	1.01–3.20	0.044	1.28	0.73–2.24	0.395
Previous BRAF + MEK inhb						
No						
Yes	1.18	0.67–2.10	0.568	1.39	0.81–2.38	0.233
Previous Chemotherapy						
No						
Yes	1.78	1.01–3.12	0.046	1.43	0.84–2.46	0.190
ICI treatment line						
1						
≥ 2	1.41	0.88–2.25	0.155	1.45	0.93–2.26	0.109
LDH level						
Normal						
Elevated	1.54	0.96–2.47	0.074	1.54	0.98–2.40	0.059
CALLY index						
High						
Low	2.57	1.57–4.22	< 0.001	2.18	1.38–3.44	0.001

Results are presented as hazard ratios (HR) with 95% confidence intervals (CI)

*CI* Confidence interval, *ECOG* Eastern cooperative oncology group, *HR* Hazard ratio, *ICI* Immune checkpoint inhibitor, *LDH* Lactate dehydrogenase, *OS* Overall survival, *PFS* Progression-free survival

### Comparison of the CALLY index with its components and other prognostic biomarkers

To assess whether the CALLY index offered superior prognostic value, we first compared it with its individual components—serum CRP, albumin, and lymphocyte count—by performing Cox regression analyses and calculating the concordance (C)-index (Table 4). The CALLY index demonstrated a higher C-index (0.624) than any of its three individual components (CRP, 0.599; albumin, 0.572; lymphocyte count, 0.607), suggesting that integrating these parameters enhances prognostic accuracy. We then expanded our evaluation to include other established prognostic biomarkers. As summarised in Table 4, the CALLY index’s C-index was comparable to or slightly higher than the values observed for PNI (0.614), mGPS (0.605) and platelet-to-lymphocyte ratio (PLR) (0.601). Although the neutrophil-to-lymphocyte ratio (NLR) showed a marginally higher C-index (0.627), the difference was minimal, indicating that the CALLY index is at least as reliable as these other markers in predicting overall survival in metastatic melanoma.

**Table 4:** Cox Regression and C-Index Results for the CALLY Index and other biomarkers

	Cutoff value	HR	95% CI	*p*	C-index
CALLY index	≤ 2	2.57	1.57–4.22	< 0.001	0.624
	> 2	Ref	Ref		
CRP, mg/dl	$\le$ 3	Ref	Ref	< 0.001	0.599
	> 3	3.73	2.10–6.63		
Albumin, g/dl	≤ 3.5	2.49	1.40–4.42	0.002	0.572
	> 3.5	Ref	Ref		
Lymphocyte /µL	≤ 1520	1.91	1.19–3.07	0.007	0.607
	> 1520	Ref	Ref		
PNI	≤ 49.4	2.28	1.40–3.73	0.001	0.614
	> 49.4	Ref	Ref		
mGPS	0	Ref	Ref	0.006	0.605
	1	1.87	1.13–3.12		
	2	3.70	1.55–5.85		
NLR	≤ 4.5	Ref	Ref	< 0.001	0.627
	> 4.5	3.33	1.97–5.61		
PLR	≤ 126	Ref	Ref	0.004	0.601
	> 126	2.34	1.32–4.17		

The C-index values were calculated to compare the predictive power of each parameter

*CI* Confidence interval, *HR* Hazard ratio, *OS* Overall survival, *PFS* Progression-free survival, *CRP* C-reactive protein, *PNI* Prognostic nutritional index, *mGPS* Modified Glasgow prognostic score, *NLR* Neutrophil–lymphocyte ratio, *PLR* Platelet–lymphocyte ratio

### Predictive nomogram for OS

**Fig. 3:**
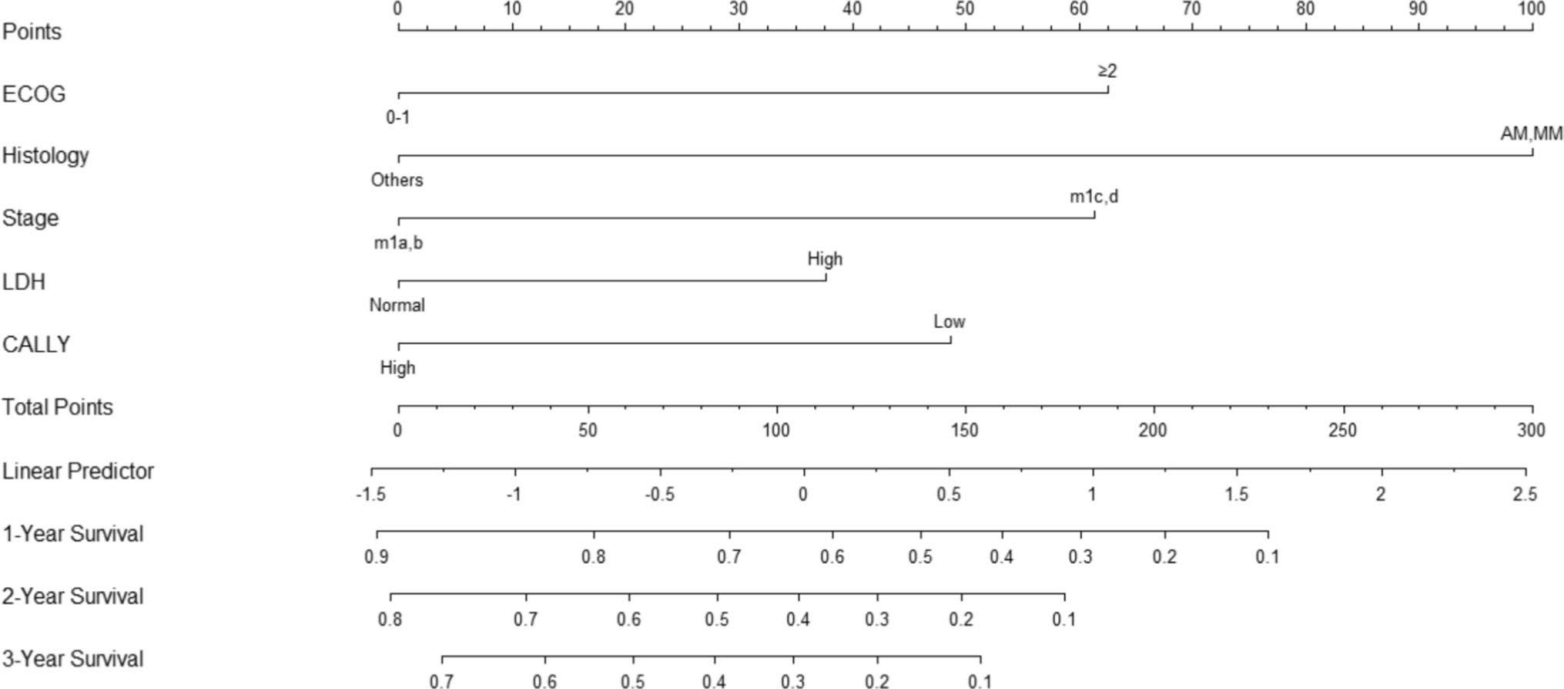
Nomogram for Overall Survival Prediction Incorporating the CALLY Index

**Fig. 4:**
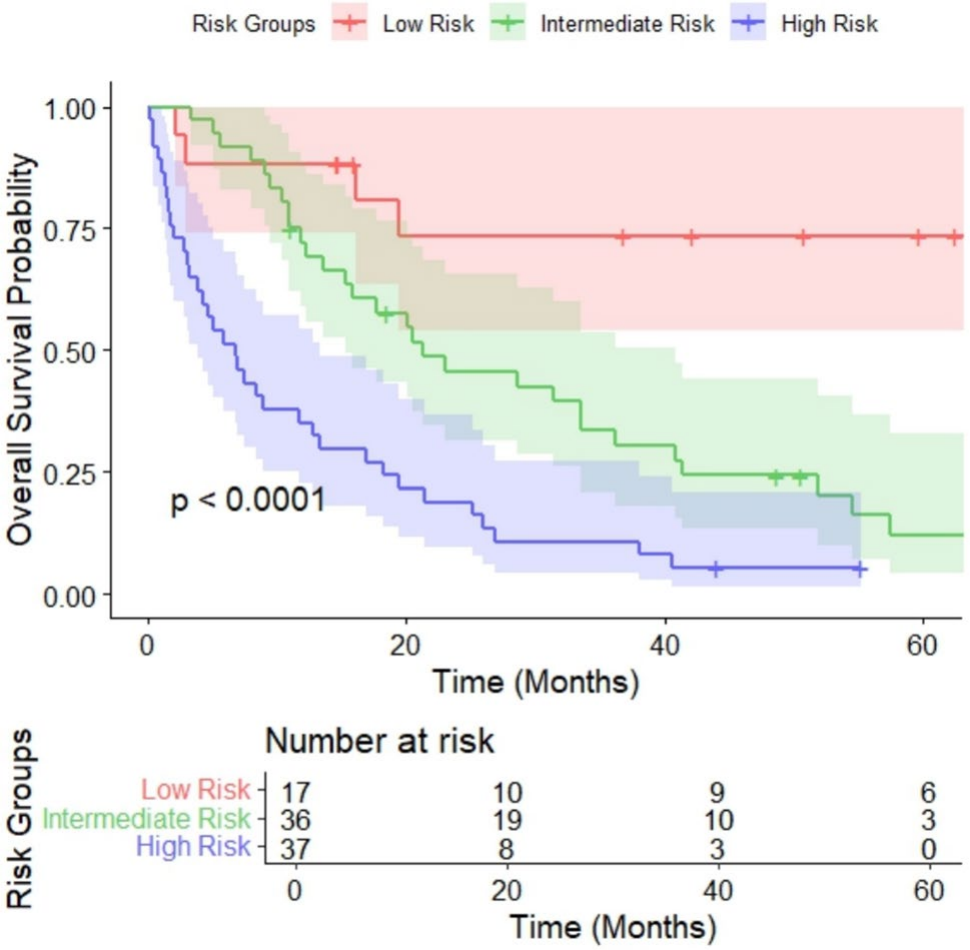
Kaplan–Meier Survival Curves and Log-Rank Test Results for Patients Stratified into Low-, Intermediate- and High-Risk Groups Based on Nomogram Scores

A nomogram was constructed using those variables identified as statistically significant in the multivariate Cox regression analysis, including LDH > ULN, M1c and M1d staging, the acral and mucosal melanoma types, a CALLY index ≤ 2 and an ECOG score ≥ 2. The nomogram is presented in Fig. 3. The C-index of the nomogram was calculated to be 0.705 (95% CI: 0.634–0.776) (based on 1,000 bootstrap resamples). Additionally, the area under the curve (AUC) values for the 12-month OS and 36-month OS were 0.767 (95% CI: 0.661–0.860) and 0.752 (95% CI: 0.646–0.846), respectively. To further assess the nomogram’s performance, calibration curves at 12, 24 and 36 months (Figures S2–S4) demonstrated good agreement between predicted and observed survival probabilities. Moreover, a decision curve analysis (Figure S5) indicated a favourable net benefit across a range of threshold probabilities, supporting the model’s potential clinical utility. Based on the Kaplan–Meier and log-rank test results, the optimal cutoff values derived from the nomogram were used to classify the patients into three risk groups: low, intermediate and high. More specifically, the cutoff points were determined as follows: low risk–intermediate risk at 37.72 and intermediate risk–high risk at 111.24. The Kaplan–Meier survival curves for the risk groups are shown in Fig. 4.

According to the Kaplan–Meier estimates, the median OS was 6.73 months (95% CI: 3.80–13.30 months) in the high-risk group and 21.30 months (95% CI: 15.23–40.9 months) in the intermediate-risk group, while it was not reached in the low-risk group.

## Discussion

To the best of our knowledge, this study is the first to define the role of the CALLY index in patients with metastatic melanoma who are treated with anti-PD-1 therapy. Our findings demonstrated that the CALLY index remains an independent prognostic factor for objective response, PFS and OS in multivariate analyses. Importantly, the CALLY index showed a higher C-index value than its individual components (i.e. the CRP, albumin and lymphocyte counts), underscoring its multifaceted prognostic utility. Indeed, the CALLY index provides a multifaceted assessment by capturing systemic inflammation via the CRP level, nutritional status through the albumin level and antitumour immunity with the lymphocyte count, thereby offering a broader perspective when compared with other inflammatory and immunonutritional indices [13].

ICI therapies primarily exert their effects by enhancing antitumour responses and creating immune memory via T lymphocytes. Consequently, lymphocytes are fundamental to the efficacy of ICIs. Prior studies have explored the relationship between the peripheral lymphocyte count and the efficacy of ICIs, showing that both the pre-treatment lymphocyte count and changes in the lymphocyte level during treatment correlate with survival in patients treated with ICIs [14, 15]. Systemic inflammation plays a pivotal role in tumour progression and metastasis, and it can also impair ICI efficacy by creating an immunosuppressive microenvironment [16]. In the context of ICI therapy, IL-6 has emerged as a key mediator of treatment resistance. IL-6 exerts immunosuppressive effects by inducing regulatory T-cells and myeloid-derived suppressor cells, thereby suppressing the antigen-presenting functions of dendritic cells and macrophages [17]. Notably, an analysis of patients with melanoma in the CheckMate-067 trial revealed that elevated IL-6 was an independent predictor of poor survival among those treated with nivolumab and ipilimumab [18]. CRP, which is produced by hepatocytes in response to IL-6 stimulation, is a cost-effective and widely available marker of inflammation that may also contribute to an immunosuppressive tumour microenvironment by directly impairing T-cell function [19, 20]. Moreover pre-treatment CRP levels have been associated with poor survival in patients with metastatic melanoma who are undergoing ICI therapy [21]. Collectively, these processes diminish the host’s antitumour immune response, ultimately leading to reduced efficacy of immune checkpoint inhibitors. Similarly, serum albumin, which is regulated by IL-6-mediated hepatic production, serves as a marker of both chronic inflammation and nutritional status [22]. Previous studies have indicated that malnutrition, alongside chronic inflammation, may impair adaptive immunity and contribute to tumour progression in patients who are receiving ICIs [9]. Moreover, hypoalbuminemia has been linked to poor treatment efficacy and lower survival rates in these patients [23]. Therefore, the CALLY index has emerged as a potential prognostic biomarker for use in patients treated with ICIs due to its ability to simultaneously evaluate immune system function, inflammation and nutritional status.

The prognostic importance of the CALLY index has been demonstrated in various tumours when it comes to the relapse-free survival and OS [24]. However, studies exploring its prognostic value in patients treated with ICIs are scarce, while no prior study has assessed its role in patients with metastatic melanoma. Previous studies have proposed various cutoff values for the CALLY index (ranging from one to five), although no such value has been validated in large cohorts [12, 13]. In the present study, the optimal cutoff value for the CALLY index was determined to be two using Kaplan–Meier and log-rank tests. Still, this value requires external validation in larger patient populations to comprehensively assess its predictive value.

While prior studies have established the prognostic importance of inflammatory indices such as the NLR, PLR, lymphocyte-to-monocyte ratio, systemic immune-inflammation index and pan-immune-inflammation value in patients with metastatic melanoma [25, 26], limited research has examined the impacts of immunonutritional indices. Among these indices, the PNI and mGPS have been shown to be prognostic in cases of metastatic melanoma [10, 27]. However, these indices often rely on fewer parameters, potentially overlooking the multifactorial interplay between systemic inflammation, nutrition and immunity. In our study, the CALLY index demonstrated prognostic performance at least comparable to PNI (C-index = 0.614), mGPS (C-index = 0.605), and PLR (C-index = 0.601) in predicting overall survival. Although NLR showed a marginally higher C-index (0.627), the difference was minimal. Our study is the first to demonstrate the association of the CALLY index, as another immunonutritional index, with the survival and treatment response in patients with metastatic melanoma who are treated with anti-PD-1.

In addition to the CALLY index, the multivariate Cox regression analysis identified the acral and mucosal type, M1c/M1d staging, an ECOG score ≥ 2 and LDH > ULN as independent predictors of OS. While the acral and mucosal types are rare in European populations (1–2%), they are more common in Asian populations (up to 40%), in whom studies suggest the lower efficacy of anti-PD-1 therapy [28]. As expected, an advanced disease stage, poor performance status and elevated LDH (a marker of the tumour burden) were also associated with poor survival [29]. Notably, these clinical and laboratory factors—particularly M1c/M1d and elevated LDH—could coincide with increased systemic inflammation [30, 31], while a poor ECOG score may reflect inadequate nutrition [32], both of which align with the CALLY index’s components. Indeed, in our baseline comparisons, LDH was significantly higher in the low CALLY group (*p* < 0.001), and stage M1c/M1d demonstrated a trend towards significance (*p* = 0.091), whereas other variables did not differ. Although the multivariate analysis confirmed the independent contributions of these variables alongside the CALLY index, larger cohorts are warranted to explore potential interaction effects more rigorously and to clarify how these overlapping factors might modify the prognostic impact of the CALLY index.

Nivolumab–ipilimumab combination therapy is recommended as a first-line treatment in patients with metastatic melanoma due to its higher efficacy. However, given the high toxicity risk associated with such treatment, identifying patient groups for whom anti-PD-1 monotherapy provides similar efficacy is a subject of interest [33]. In this regard, our study demonstrated an independent association between the CALLY index and OS, leading to the development of a nomogram that incorporates additional independent predictors identified through a multivariate Cox regression analysis. The subsequent Kaplan–Meier and log-rank analyses enabled the stratification of the patients into three risk groups: low, intermediate and high. Significant differences in the OS were observed among these three groups (*p* < 0.001). Notably, in the low-risk group, longer survival durations were achieved with anti-PD-1 monotherapy, suggesting that combination ICI therapy may not be necessary for these patients. Conversely, the high-risk patients exhibited shorter survival durations with anti-PD-1 therapy, indicating that they may benefit more from combination ICI therapy. Although these findings highlight the potential of the CALLY index-based nomogram to guide treatment selection, we did not directly compare monotherapy with combination immunotherapy in this study. Consequently, larger, multicentre validations—including head-to-head comparisons of monotherapy versus combination therapy across distinct risk strata—are needed to determine whether risk-tailored strategies based on the CALLY index can effectively guide ICI treatment selection.

This study has several limitations. First, as a retrospective, single-centre analysis with a relatively small sample size, the findings may not be generalisable to broader populations. The limited cohort also reduced our statistical power and prevented validation of the new CALLY index cutoff and nomogram in an independent dataset. Second, because no prior studies have examined the CALLY index in melanoma, our cutoff was derived from the same cohort, emphasising the need for external validation in larger populations. Lastly, multiple univariate analyses were performed without formal adjustments for multiple comparisons, as these were exploratory in nature.

## Conclusion

This study is the first to demonstrate the prognostic value of the CALLY index in patients with metastatic melanoma who are treated with anti-PD-1 therapy. The CALLY index, due to integrating markers of systemic inflammation, nutritional status and immune status, serves as an independent prognostic factor in terms of the objective response, PFS and OS. The developed nomogram, which incorporates the CALLY index and other significant variables, effectively stratifies patients into distinct risk groups with differing survival outcomes.

Given its cost-effectiveness and accessibility, the CALLY index is a practical tool that clinicians can use in predicting survival outcomes and tailoring treatment strategies for patients with melanoma who are undergoing ICI therapy. However, external validation in larger cohorts is essential to establish its broader applicability and confirm he optimal cutoff value determined in this study. Future research should focus on prospective validation studies and the standardisation of the CALLY index assay, which would facilitate consistent clinical implementation.

## Supplementary Information

Below is the link to the electronic supplementary material.

Supplementary file1 (DOCX 606 KB)

## Data Availability

The datasets used and/or analysed during the current study are available from the corresponding author on reasonable request.
