# Supplementary material for: Prognostic utility of the CALLY index in metastatic melanoma: building a nomogram for Patients on Anti-PD-1 therapy
Source: Clin Transl Oncol. 2025 Mar 16;27(9):3770–80. doi: 10.1007/s12094-025-03888-z (PMC12399701; doi:10.1007/s12094-025-03888-z)
Supplement: Supplementary file 1 — Supplementary file1 (DOCX 606 KB) [file 12094_2025_3888_MOESM1_ESM.docx]

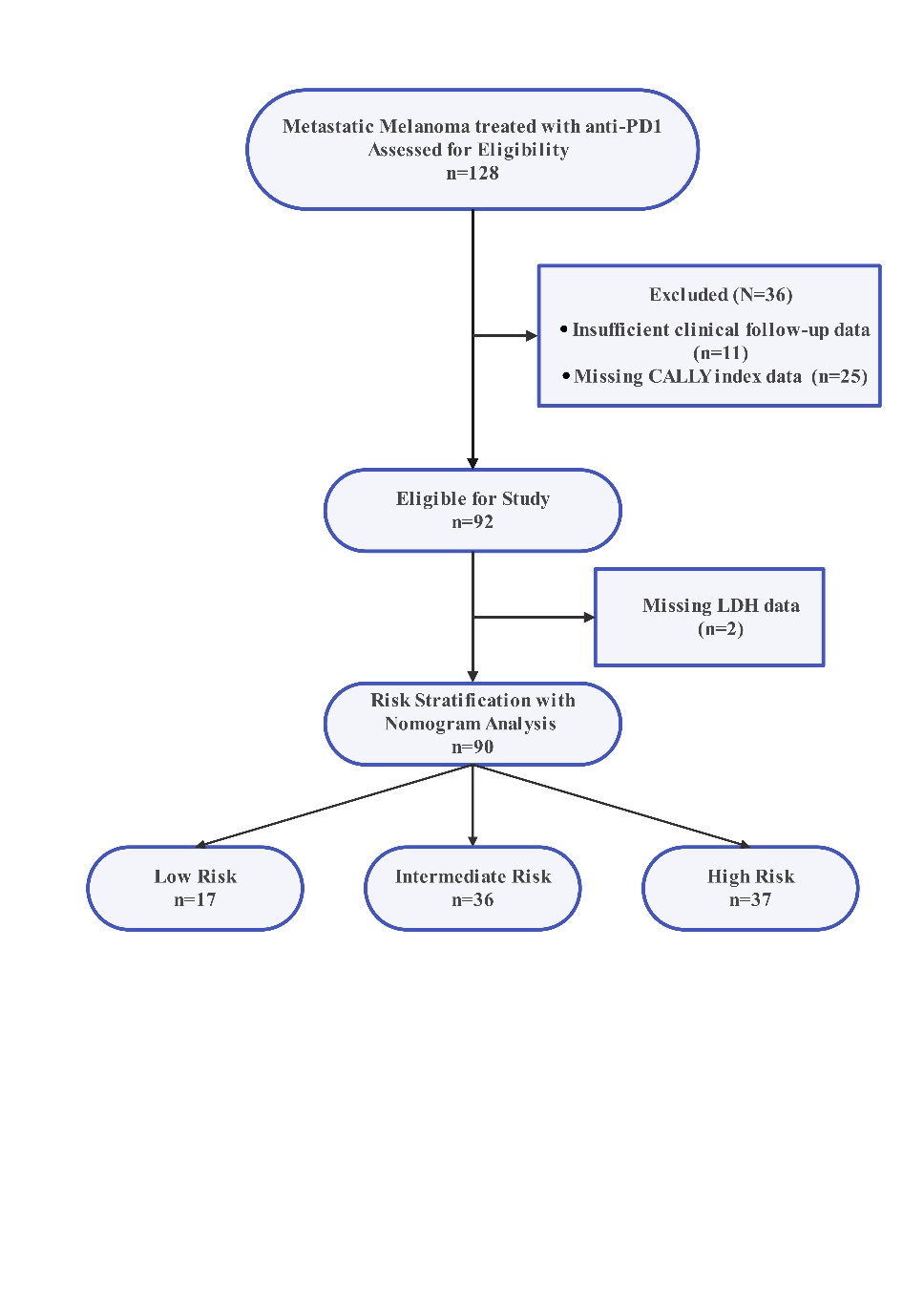


Figure S1. A flow chart illustrating the inclusion of patients


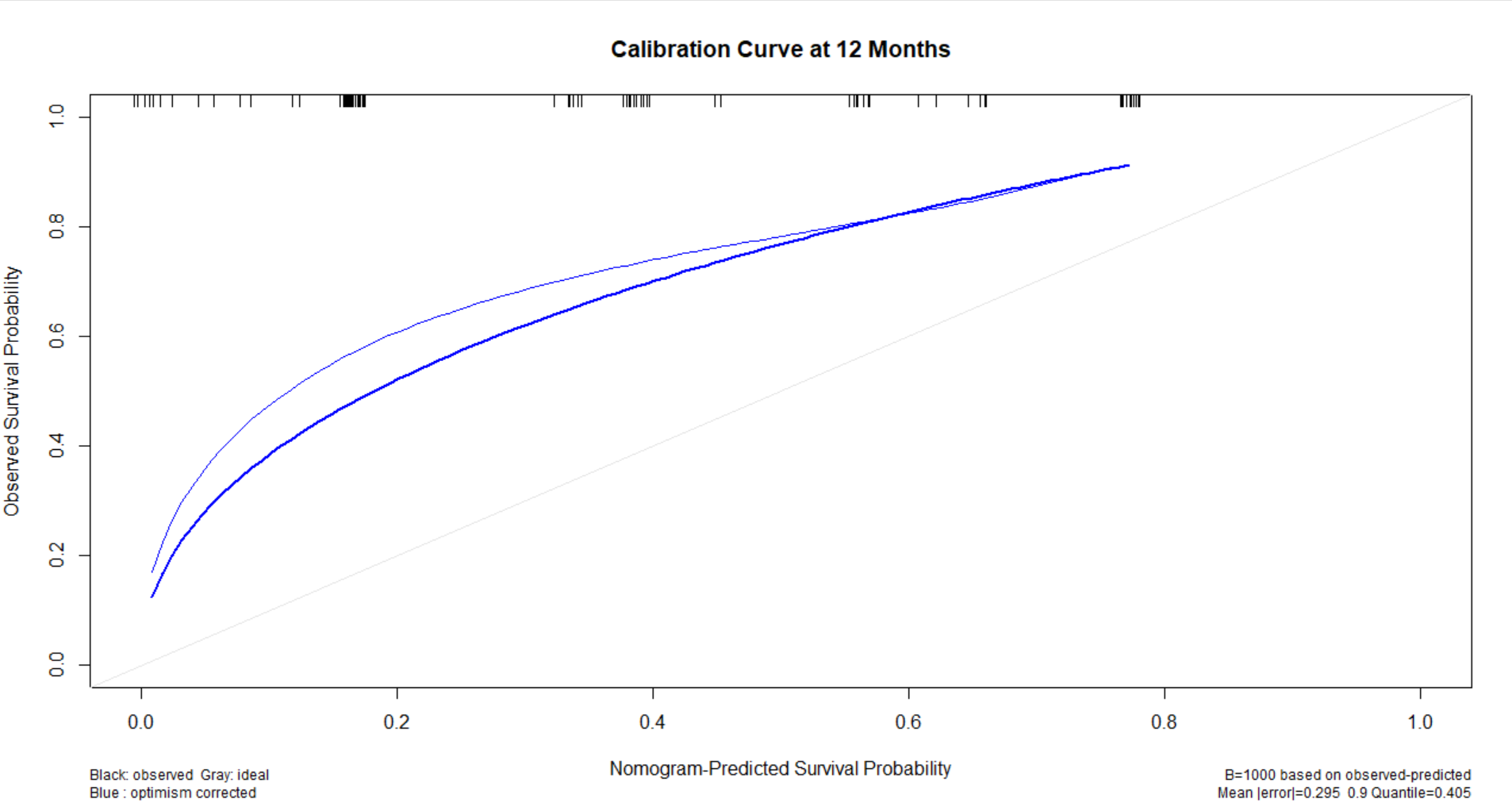


Figure S2. Calibration curve at 12 months


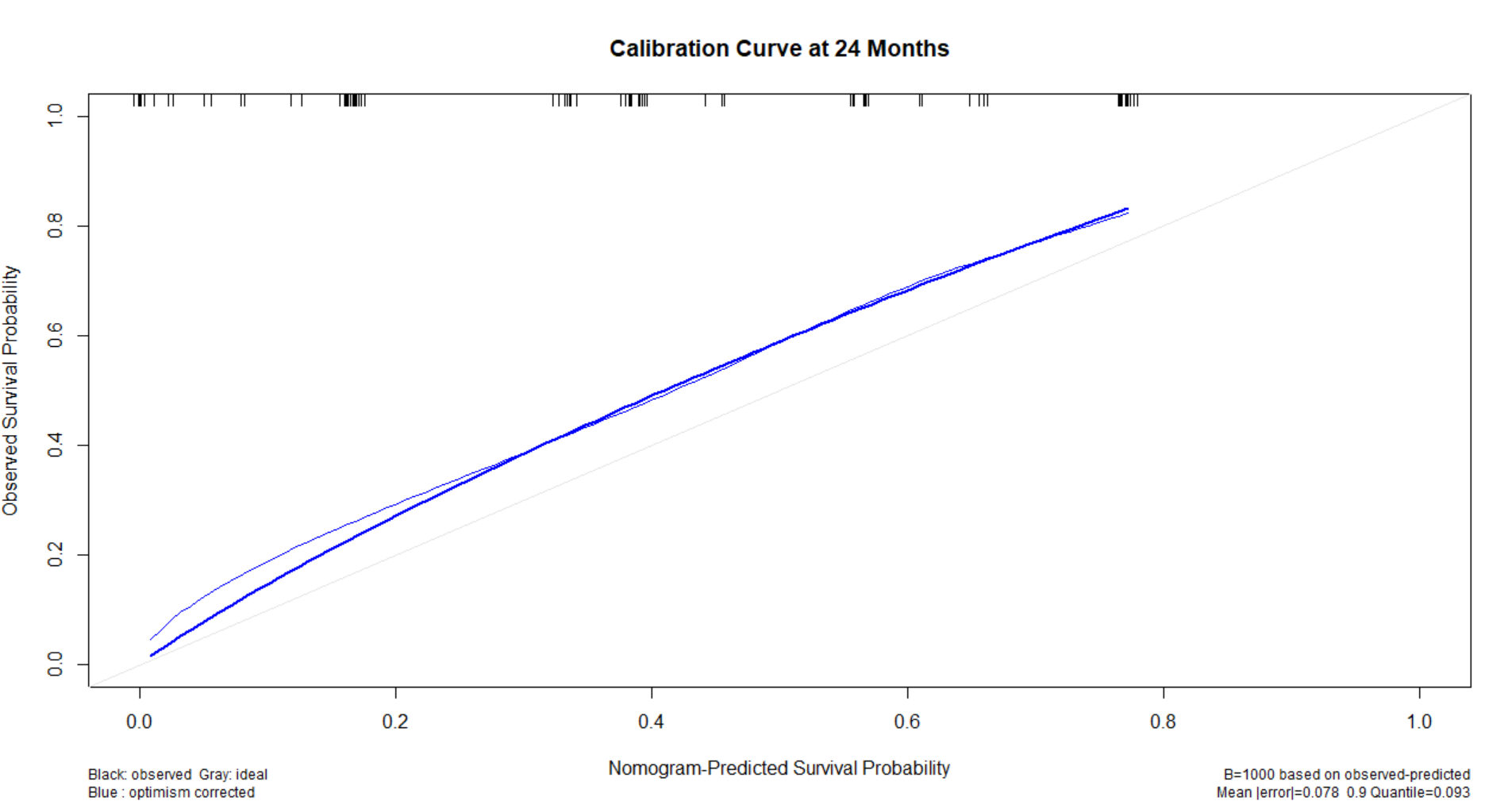


Figure S3. Calibration curve at 24 months


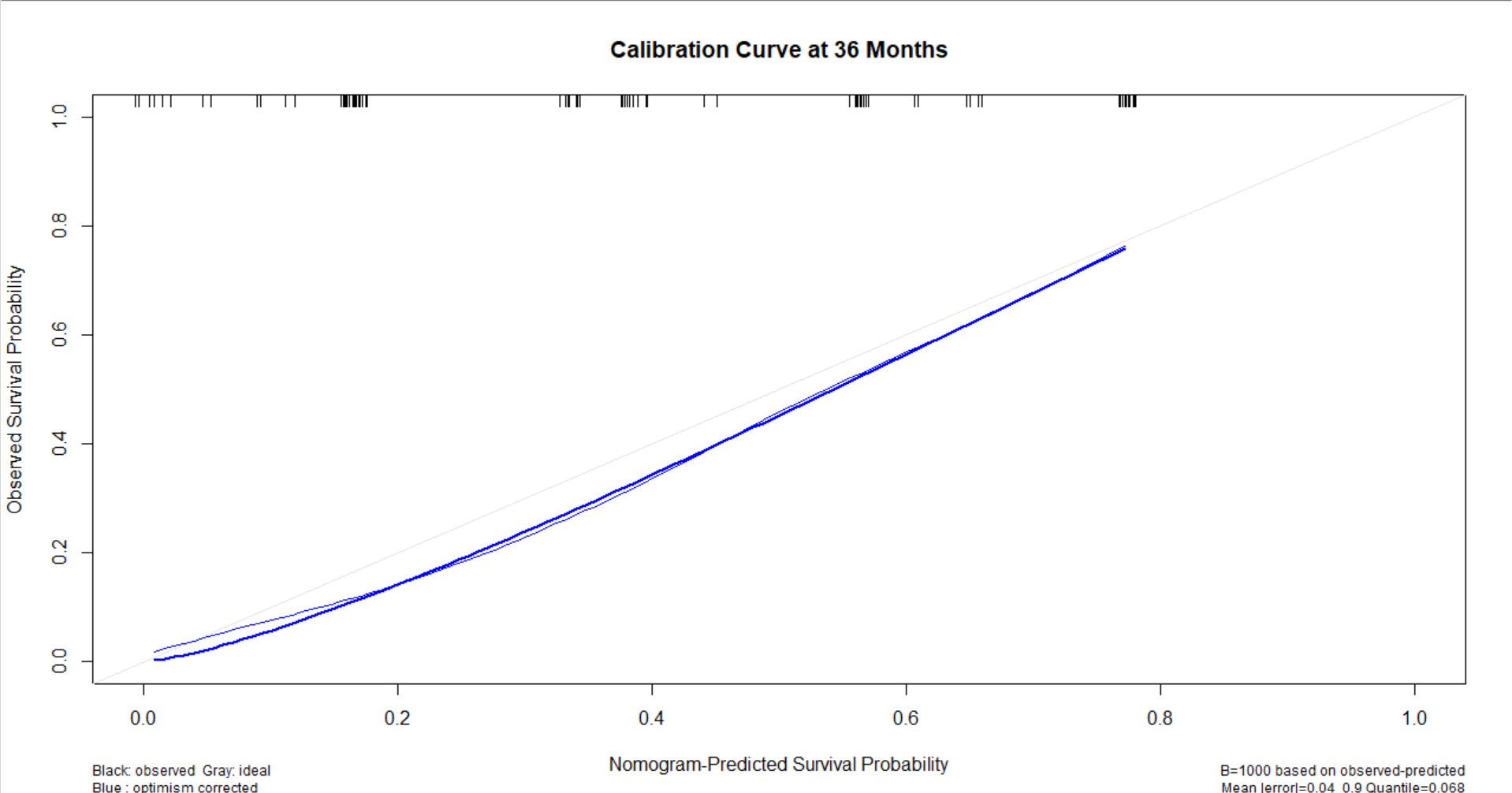


Figure S4 Calibration curve at 36 months


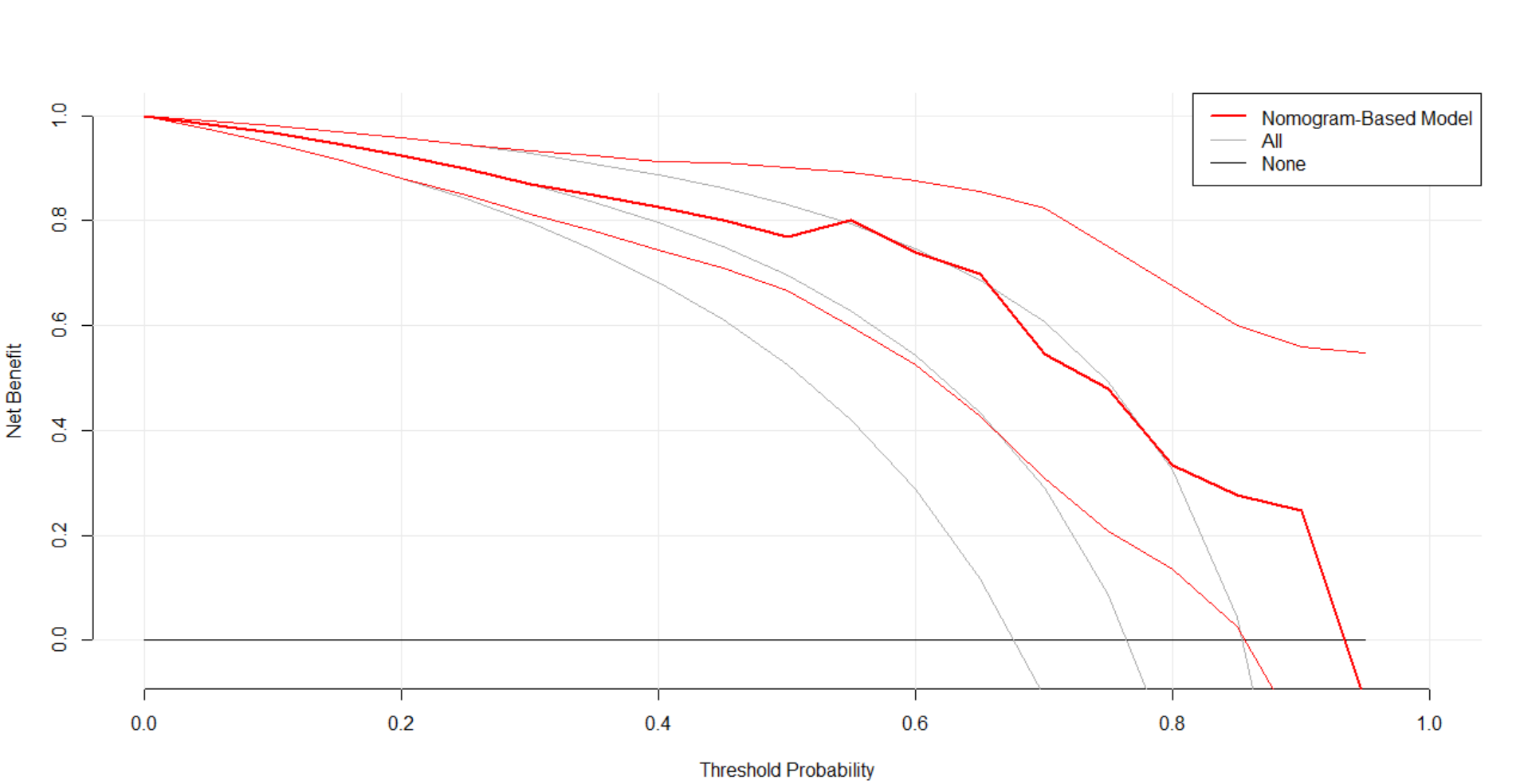


Figure S5. Decision curve analysis for the nomogram-based model in predicting clinical outcomes.
